# Food Safety and Management System Audits in Food Retail Chain Stores in Greece

**DOI:** 10.3390/foods13030457

**Published:** 2024-02-01

**Authors:** Michalis Psomatakis, Konstantinos Papadimitriou, Andreas Souliotis, Eleftherios H. Drosinos, Georgios Papadopoulos

**Affiliations:** 1 Department of Food Science and Human Nutrition, Agricultural University of Athens, 11855 Athens, Greece; psomatesmih@gmail.com (M.P.); ehd@aua.gr (E.H.D.); 2 Department of Nutrition and Dietetics, Harokopio University of Athens, 17676 Athens, Greece; andreassouliotis@yahoo.com; 3 Institute for Design and Analysis of Experiments, University Research Center, Agricultural University of Athens, 11855 Athens, Greece; gpapadop@aua.gr

**Keywords:** audit, food safety management system, HACCP, food retail stores

## Abstract

The present study aimed to assess the performance of food safety management systems in food retail stores via audits to reveal potential areas of improvement and to find out possible corrective actions to suggest to the top management. Two cycles of on-site audits took place in 106 stores to assess the requirements and hygiene conditions. After the first cycle of audits, improvements were suggested to the top management, and a second cycle of audits took place after a reasonable time. In the checklist, we recorded the temperatures of retail refrigerators and the scores from the inspection of hygiene and HACCP documentation. In the A′ audit, the percentage of stores that had higher temperatures than the critical limits was equal to 51%, and those temperatures occurred in the refrigerators for salads, followed by the refrigerators for deli meat, yogurts and desserts. In the B′ audit, only the refrigerators for salads exhibited percentages that were statistically significant lower (*p*-value < 0.05), and the stores were improved after the audit. High percentages of high-scoring stores were observed in the A′ and B′ audit in the inspection of HACCP documentation, although there was not a statistically significant improvement observed (*p*-value > 0.05). In the hygiene inspection, statistically significant improvement with 95% confidence appeared for “Refrigerator’s products appearance”, “Storage cleanliness”, and “Grocery shelf cleanliness”. The highest number of non-conformities without statistically significant improvement was found for “Checking temperatures of the receiving products” and “Labeling of fruit store products”, with the percentages being lower than 15% in both of the audit cycles. Many employees of the stores did not check and record the temperatures of receiving products from suppliers. In addition, the storage of spoiled products beneath fresh products for selling in the same refrigerator is not a good practice. Greater efforts must be made by top management and employees to maintain and distribute food products in the best and safest possible hygiene conditions.

## 1. Introduction

It has been sufficiently proven that the effective application of the Hazard Analysis and Critical Control Points (HACCP) system has plenty of benefits and significant positive results in food safety [1,2,3,4,5,6]. Food business operators (FBOs), according to Regulation (EC) No. 852/2004 [7], have implemented the HACCP system as compulsory. In addition, certain FBOs implement voluntary standardized food safety management systems (FSMSs) based on international food safety standards and certification schemes such as the British Retail Consortium Global Standard [8], the International Featured Standard [9], the International Organization for Standardization 22000 [10], and the Food Safety System Certification 22000 [11]; this is performed in order to not only control their processes but also improve the satisfaction of their customers [4,12]. It has been noticed that the food businesses (FBs) that have certified FSMSs or implement a HACCP system have better microbiological safety performance [2,3,13]. In addition, many studies have indicated that the FBs that implement a certified FSMS had a significantly higher average score in the performance of the systems compared with the non-certified ones [12,14,15,16,17]. Food safety is not only dependent on the efficiency of FSMSs, but also on the characteristics of the context where they operate.

In 2021, there were more than 32,000 cases of illness coming from foodborne outbreaks in the European Union (EU) and United Kingdom, 31 of which were lethal, and there was a 29.8% increase in foodborne outbreaks compared with the previous year. In addition, the hospitalizations also increased compared with the year 2020 by 62.6% and 49.0%, respectively. However, the number of reported deaths decreased by 8.8%. Of the 355 total outbreaks reported in the EU, 34.1% were connected to the category of restaurants, pubs, street vendors, take-out, etc. [18]. This percentage in food services and food retailers may be associated with low compliance to good hygiene practices (GHPs) by the employees or improper storage conditions [19,20,21,22,23,24,25,26]. Small and medium-sized enterprises (SMEs) are confronted with various difficulties and barriers in establishing and implementing an effective FSMS. This is mostly due to the lack of resources and financial limitations [1,27,28].

As retailers are part of the food chain, they contribute to safety. This study aimed to assess the performance of FSMSs in food retail chain stores via audits and to reveal potential opportunities for improvement. Our study provides insight into the major non-conformities and possible corrective actions for this type of organization.

## 2. Materials and Methods

### 2.1. Food Retail Stores and Audits

One hundred and six (106) SME food retail stores of a well-known chain located in Attica (Greece) were selected for the audits. The food products that are available in these stores are always received pre-packed. On-site audits took place to assess the fulfilment of food safety requirements and the hygiene conditions of the stores using a checklist in two independent cycles. After the first audit, corrective actions were recommended based on the analysis and evaluation of the initial findings. A reasonable period was given to the audited stores in order to comply, and then the second cycle of audits took place. The study was carried out in a two-year period (2019–2020). The A′ audit took place from 18 November 2019 to 24 December 2019; the B′ audit took place from 17 February 2020 to 26 March 2020. All audits were preannounced and were conducted with the approval of the top management of the chain stores.

### 2.2. The Checklist

The checklist was proposed by the quality assurance management department, and it is the one that is typically used in internal audits in this food retail chain stores. Basically, it was originally prepared according to the national hygiene requirements of the operation of food/beverage companies, European Regulation (EC) No. 852/2004 [7], and the national and European labelling Regulation (EC) No. 1169/2011 [29] in order to fulfill statutory and regulatory requirements. The checklist was initially applied in the audits we performed in ten stores, and since no unambiguity was observed it was applied throughout this study. The sections of the checklist were: 1. refrigerator temperature recording, 2. HACCP documentation (“Personnel certificates”, “Cleaning and disinfection”, “Water supply bill”, “Pest control”, “Temperatures recording”, and “Suppliers file”), and 3. hygiene inspection. The temperature of the products was taken from the refrigerator displays or the internal control thermometers (digital or mercury). In each audit, the temperature was measured in all refrigerators of the store: retail refrigerators, fruit refrigerators, and salad refrigerators. The scale of each audit criterion varied depending on the severity and levels of conformity. The highest score was “two”, meaning fulfillment of the requirements, followed by “one” and “zero”, corresponding to substantial and inadequate fulfillment of the requirements, respectively. In certain audit criteria, the scale was “one” and “zero” for full and inadequate fulfillment, respectively.

### 2.3. Statistical Analysis

An explanatory data analysis was conducted to assess the distribution of the resulting data samples. Statistical tests of the percentage of stores that had higher temperatures than the critical limits for the refrigerator products were performed to assess the improvement after the first audit and the implementation of the corrective actions (z-test for comparing two independent binomial proportions). The same statistical test was performed for the percentage of the stores that received the highest scores for the fulfillment of the requirements. Significance was identified when *p* < 0.05. A cluster analysis was used to group the stores, refrigerator temperatures, and requirements according to the degree of fulfillment. All tests were performed using Statgraphics Centurion XVII software.

## 3. Results

### 3.1. Distributions of the Different Refrigerator Temperatures in the Stores

**Figure 1:**
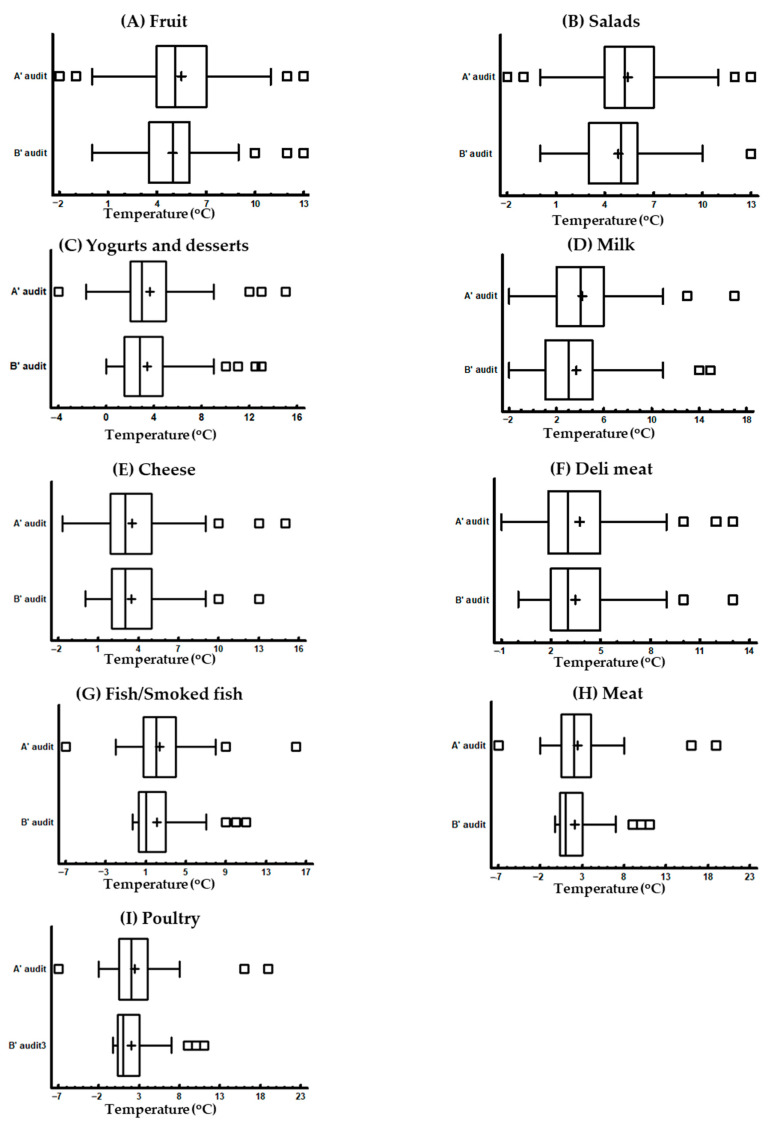
Distributions of refrigerators’ temperatures during the A′ and B′ audit, presented as box and whiskers plots. (**A**) Fruit refrigerator, (**B**) salad refrigerator, (**C**) yogurts and desserts refrigerator, (**D**) milk refrigerator, (**E**) temperatures of cheese refrigerator, (**F**) deli meat refrigerator, (**G**) Fish/smoked fish refrigerator, (**H**) meat refrigerator, and (**I**) poultry refrigerator.

In order to check the refrigerator temperatures, the critical limits of temperatures as specified in the guidelines of the Hellenic Food Authority (EFET) for food retailers were used [30]. The distribution of refrigerators’ temperatures audited are presented in Figure 1 and the relevant statistical analysis is shown in Table 1.

The median temperatures were within the limits of the critical temperatures for each product in both audits. During the A′ audit, in 50% of the stores, the range of refrigerators’ temperature values around the median (interquartile range—IQR) was 3.5 °C at the highest and 3 °C at the lowest (Table S1). In the B′ audit, the corresponding values were 3.25 °C at the highest and 2.5 °C at the lowest (Table S1). Only for the milk refrigerator were the values slightly higher at 4 °C for both audits. In the A′ audit, the highest temperature recorded was 19 °C and occurred for the refrigerators of meat or poultry (Figure 1H,I, Table S1). The highest number of outliers of high temperatures were three, and they occurred for the refrigerators of yogurts and desserts, cheese, and deli meat (Figure 1C,E,F). In the B′ audit, the highest temperature of 15 °C was found in the milk refrigerator (Figure 1D). The yogurts and desserts refrigerator had four outliers of high temperatures (Figure 1C), followed by the refrigerators of fruit, fish/smoked fish, meat, and poultry, which exhibited three outliers each (Figure 1A,G,H,I). In the A′ audit, 51% of the stores had higher temperatures in the salad refrigerators than their critical limits. The relevant percentages in deli meat and yogurts and desserts refrigerators were 48% and 44%, respectively. The remaining percentages of stores with refrigerators exceeding the appropriate temperatures were lower and ranged between 15% and 20%. In the B′ audit, the number of stores with salad refrigerators that exceeded the critical temperature of 4 °C was 39%, which was significantly lower compared with the 51% of the A′ audit (*p*-value = 0.046). In most cases, the percentages of stores with refrigerators of different foods with temperatures higher than the critical were reduced in the B′ audit compared with the A′ audit, but these differences were not significant (*p*-value > 0.05).

### 3.2. HACCP Documentation and Hygiene Inspection Scores of the Stores

**Figure 2:**
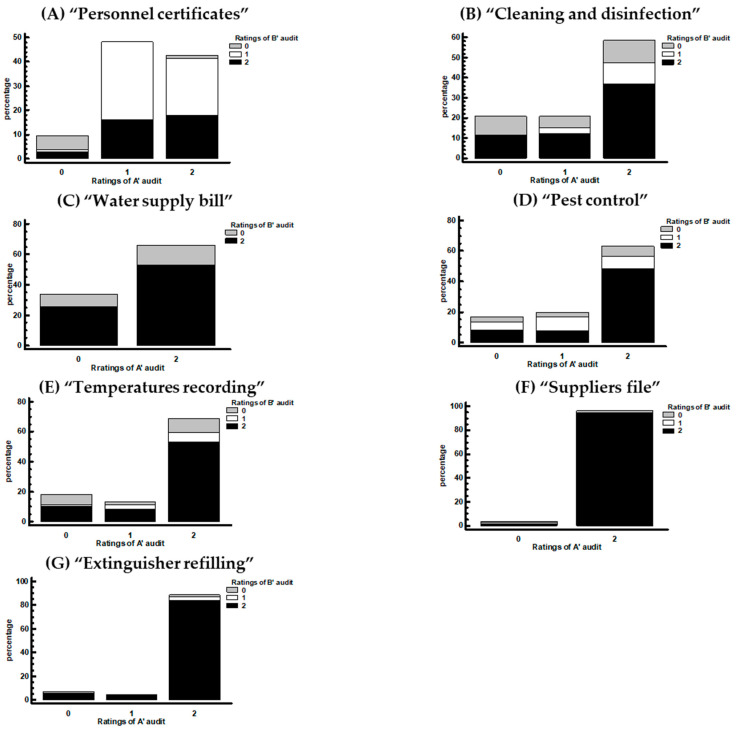
Stacked bar chart for the A′ audit by B′ audit for the HACCP documentation inspection. (**A**) “Personnel certificates”, (**B**) “Cleaning and disinfection”, (**C**) “Water supply bill” file, (**D**) “Pest control”, (**E**) “Temperatures recording”, (**F**) “Suppliers file”, and (**G**) “Extinguisher refilling”. In these figures, we can directly assess the ratings of the A′ audit and simultaneously present any change in the ratings during the B’ audit. For example, in Figure 2A, we can see that from the 43% of stores with a score of one for Audit A’, most of them obtained a score of one for audit B’ as well, while a smaller percentage obtained a score of two and none scored zero.

The score stacked bar chart for the A′ and B′ audit of the HACCP documentation inspection are presented in Figure 2A–G. In the A′ audit, the stores that had the highest scores for the HACCP documentation records ranged from 58 to 96% (Figure 2B–G). These stores received high scores for the requirements “Cleaning and disinfection”, “Temperature recording”, “Water supply bill”, “Suppliers file”, “Pest control”, and “Extinguisher refilling”. Several stores that had high scores (43%) received a high score for “Personnel certificates” (Health certificate and HACCP training certificate) (Figure 2A) requirement. In the B′ audit, the percentages of the high-score stores fluctuated and ranged from 60 to 96% (Figure 2B–G). Furthermore, the percentage of the stores with high scores for “Personnel certificates” decreased to 37% (Figure 2A).

**Figure 3:**
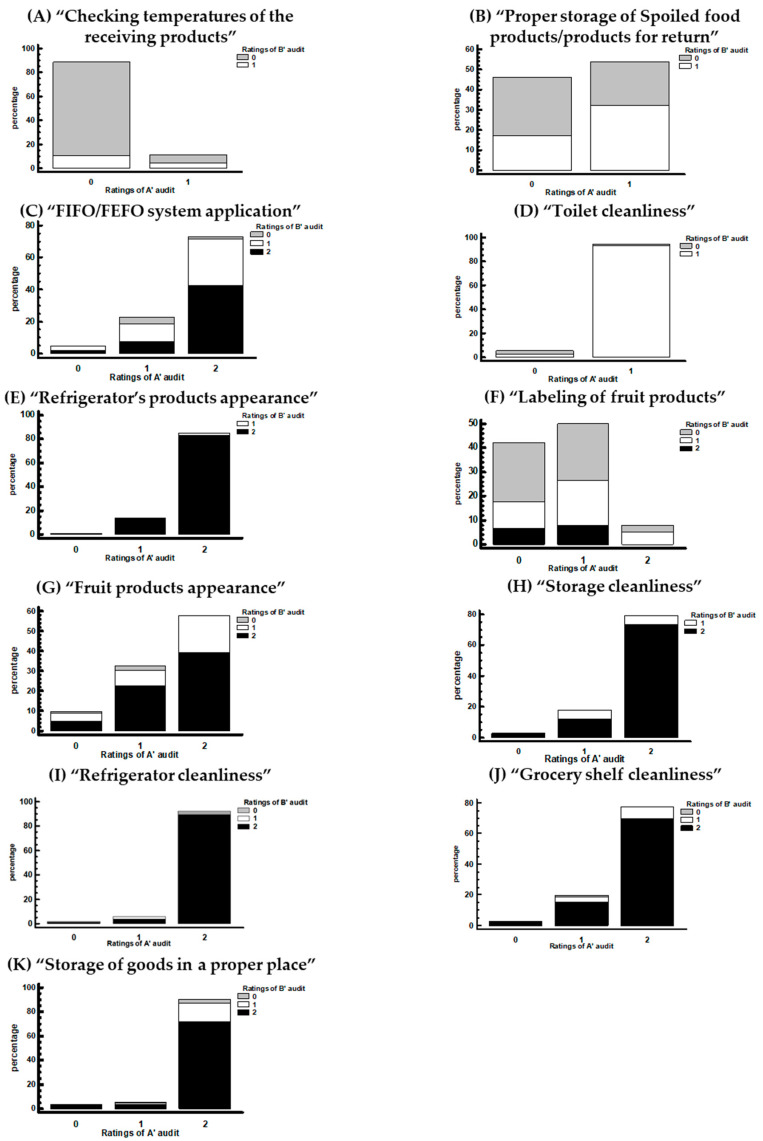
Stacked bar chart for the scores of the A′ audit by B′ audit for the hygiene inspection. (**A**) “Checking temperatures of the receiving products”, (**B**) Proper storage of “Spoiled food products/products for return”, (**C**) “FIFO/FEFO application”, (**D**) “Toilet cleanliness”, (**E**) “Refrigerator’s products appearance”, (**F**) “Labeling of fruit store products”, (**G**) “Fruit store products appearance”, (**H**) “Storage cleanliness”, (**I**) “Refrigerator cleanliness”, (**J**) “Grocery shelf cleanliness”, and (**K**) “Storage of goods in a proper place”.

The score stacked bar chart of the A′ and B′ audits of hygiene inspection are presented in Figure 3. In the A′ audit, the percentage of stores with high scores for hygiene inspection varied between 54% and 94%. The important criteria for these stores were “Proper storage of Spoiled food products/products for return”, “first in first out/first expired first out “(FIFO/FEFO) system application”, “Toilet cleanliness”, “Refrigerator’s products appearance”, “Fruit products appearance”, “Storage cleanliness”, “Refrigerator cleanliness”, “Grocery shelf cleanliness”, and “Storage of goods in a proper place” (Figure 3B–E,G–K). Despite the fact that some of these percentages of high-scored stores increased in the B′ audit, only three requirements with statistically significant improvement (*p*-value < 0.05) were found. These three requirements were the “Refrigerator’s products appearance”, the “Storage cleanliness”, and the “Grocery shelf cleanliness”, which increased from 85% to 98%, 79% to 89%, and 77% to 88%, respectively (Figure 3E,H,J). Nevertheless, some requirements presented lower percentages at the high-scored stores in the B′ audit. For the requirements of “FIFO/FEFO system application”, the percentages decreased from 73% to 52% (Figure 3C) and the “Storage of goods in proper place” decreased from 91% to 78% (Figure 3K), with a statistically significant decrease (*p*-value < 0.05). Similar results were found in the requirements of “Proper storage of spoiled products” (from 54% to 49%), but this difference was not statistically significant (Figure 3B). For the requirement of “Checking temperatures of the receiving products”, only 11% and 15% of stores were found to be in compliance during the A′ and B′ audits, respectively (Figure 3A). Concerning the “Labeling of fruit store products”, only 8% of the stores had the highest scores in the A′ audit and 14% in the B′ audit (Figure 3F). However, both of these potential improvements were not statistically significant (*p*-value > 0.05).

### 3.3. Classification of Stores, Refrigerators, and Audit Criteria

#### 3.3.1. Cluster Analysis of the Different Refrigerators Temperatures

**Figure 4:**
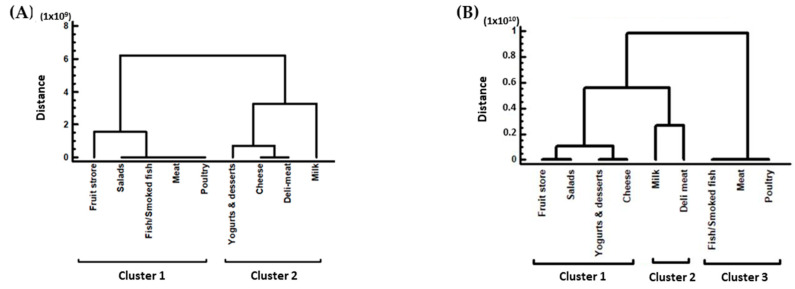
Clusters of the refrigerators of the stores. (**A**) In the A′ audit; (**B**) in the B′ audit. The dendrogram was constructed with the Ward’s method using squared Euclidean distances.

The refrigerator’s temperatures were distributed into two clusters in the A′ audit (Figure 4A). Cluster 1 consisted of the refrigerators of fruit store products, salad, fish/smoked fish, meat, and poultry and cluster 2 consisted of yogurts and desserts, cheese, deli meat, and milk. In the B′ audit, there were three clusters (Figure 4B): cluster 1 consisted of the refrigerators of fruit store products, salads, yogurts and desserts, and cheese; cluster 2 consisted of milk and deli meat refrigerators; and cluster 3 consisted of fish/smoked fish, meat, and poultry refrigerators. The extra cluster determined from the refrigerator’s temperatures in the B′ audit indicated a greater diversity of the temperatures for this audit.

#### 3.3.2. Cluster Analysis of the Stores Based on the Refrigerator Temperatures

**Figure 5:**
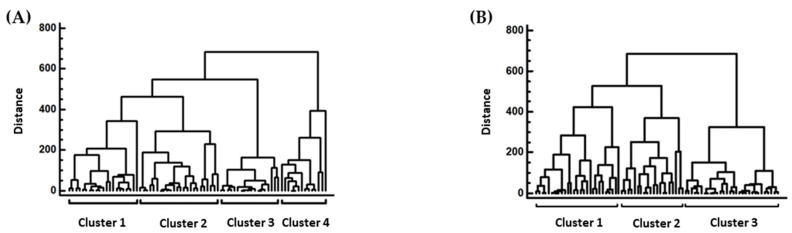
Clusters extracted from stores concerning the refrigerator temperatures. (**A**) In the A′ audit; (**B**) in the B′ audit. The dendrogram was constructed with the Ward’s method using squared Euclidean distances.

The stores in the A′ audit formed four clusters (Figure 5A) and in the B′ audit, they formed three (Figure 5B) clusters according to the temperatures of their refrigerators. In the B′ audit, the reduction of clusters indicates that the stores had a more uniform distribution of temperatures than the ones in the A′ audit.

#### 3.3.3. Cluster Analysis Based on Scores of Hygiene Inspection

**Figure 6:**
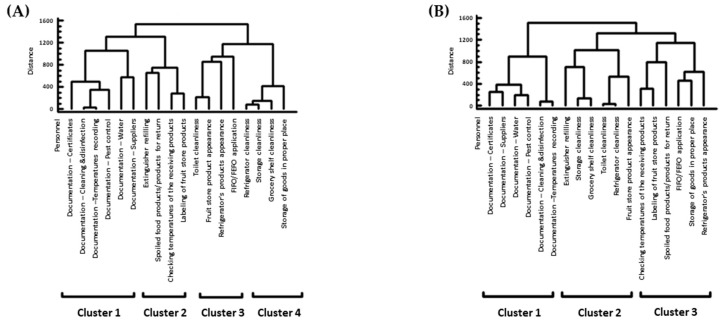
Clusters extracted from items concerning scores of hygiene inspection: (**A**) in the A′ audit; (**B**) in the B′ audit. The dendrogram was constructed with the Ward’s method using squared Euclidean distances.

**Figure 7:**
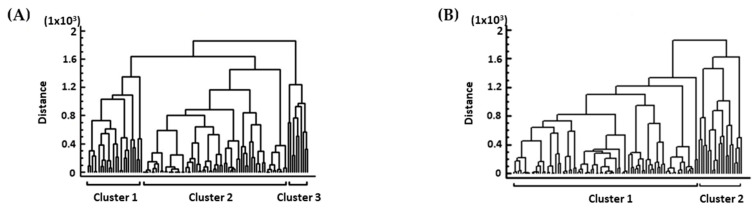
Clusters extracted from stores concerning scores of hygiene inspection. (**A**) in the A′ audit; (**B**) in the B′ audit. The dendrogram was constructed with the Ward’s method using squared Euclidean distances.

The scores of the hygiene criteria were distributed into four clusters in the A′ audit (Figure 6A). Cluster 1 consisted of documentation for: “Personnel certificates”, “Cleaning and disinfection”, “Temperatures recording”, “Pest control”, “Water supply bill”, and Suppliers”. Cluster 2 consisted of “Extinguisher refilling”, “Spoiled food products/products for return”, “Checking temperatures of the receiving products”, and “Labeling of fruit store products”. Cluster 3 consisted of “Toilet cleanliness”, “Fruit store products appearance”, “Refrigerator’s products appearance”, and “FIFO/FEFO application”. Cluster 4 consisted of “Refrigerator cleanliness”, “Storage cleanliness”, “Storage of goods in proper place”, and “Grocery shelf cleanliness”. The number of the clusters in the B′ audit was three (Figure 6B). Cluster 1 consisted of documentation for “Personnel certificates”, “Suppliers”, “Water supply bill”, “Pest control”, “Cleaning and disinfection”, and “Temperatures recording”. Cluster 2 consisted of “Extinguisher refilling”, “Storage cleanliness”, “Grocery shelf cleanliness”, “Toilet cleanliness”, “Refrigerator cleanliness”, and “Fruit store products appearance”. Cluster 3 consisted of “Checking temperatures of the receiving products”, “Labeling of fruit store products”, “Spoiled food products/products for return”, “FIFO/FEFO application”, “Storage of goods in proper place”, and “Refrigerator’s products appearance”. Moreover, the stores according to the scores of the hygiene criteria in the A′ audit were distributed in three clusters and in two clusters in the B′ audit (Figure 7A,B). The lower number of clusters in the B′ audit indicates once more that the stores had a diminished diversity of scores than those in the A′ audit.

## 4. Discussion

In the present study, two consecutive audits were conducted in SME stores of the same retail chain to verify the efficiency of the FSMS applied and assess whether corrective actions suggested in between the audits resulted in quantifiable improvements. Food safety culture is a prerequisite for an effective implementation of any FSMS. Food safety culture is directly related with leadership commitment, employee engagement, communication, accountability and responsibility, continuous improvement, and risk awareness.

Although many studies have mentioned the importance and the numerous benefits of implementing FSMS in order to ensure the food safety in FBs [1,2,4,5,6,27], the results of the present study indicated a heterogeneous trend in the implementation of FSMSs in the food retail sector, even after suggested corrective actions and audits. While some stores demonstrated improvement, others showed no change or even a decline. Our analysis revealed that only certain requirements, such as refrigerated salad temperatures, “Refrigerator product appearance”, “Storage cleanliness”, and “Grocery shelf cleanliness”, showed statistically significant improvement. On the other hand, the stores showed a decline in the requirements for “Proper storage of goods” and application of the FIFO/FEFO system. The median temperatures of fish/smoked fish products were within the critical regulated limits of the temperatures in both audits. Similar results with slightly lower average temperatures were found in fish products [31]. However, inappropriately maintained temperatures were noticed in fresh and processed fish packed in vacuum that exceeded more than 1 °C the critical temperatures [23], reaching aberrations of even >3 °C [22]. Similar results were noticed in ready-to-eat (RTE) seafood products that had higher temperatures than the manufacturer’s recommendations [32].

In this study, the median temperatures of meat, deli meat, and poultry products were within the critical limits of the temperatures for the products in both audits. However, 48% in the A′ audit and 44% in the B′ audit of the stores had temperatures > 3 °C for the refrigerators of deli meat products. Similar results were found in products of ham, with an average temperature at 2.8 °C [33] and meat products and fresh meat at 1.3 °C and 4.5 °C [22].

In the present research, the mean temperatures of yogurts and desserts, milk, and cheese were within the proper storage temperatures. However, the refrigerators of yogurts and desserts of 44% in the A′ audit and 38% in the B′ audit of stores had temperatures that were higher than the critical ones (3 °C). Appropriate temperatures were also recorded for dairy products that took place in Greece and Spain with an average temperature of 5 °C and 4 °C, respectively [31,34]. Nevertheless, in another study about different types of cheese the average temperatures were higher than the proper ones and ranged from 7.3 °C to 8.8 °C [35].

The median refrigerator temperatures of fruit store and salads were within the critical limits. However, in the refrigerator of salads, 51% in the A′ audit and 39% in the B′ audit of stores had a temperature higher than 5 °C. The storage temperature at retail and at home, the duration of storage, and the serving size are the most considerable factors for the risk of listeriosis from the consumption of leafy greens, which are intended to be eaten raw [36]. The proper storage temperature for leafy greens is 5 °C for food safety. Temperatures higher than 5 °C have been found in most of the stores for the leafy green products [19]. The widely used open-type refrigerators in retail stores do not have the required energy efficiency and often do not provide appropriate storage temperatures for packaged fresh fruits and vegetables [37].

Many authors indicate the importance of maintaining various products at the proper temperatures since the growth of *Listeria monocytogenes* is possible, even at low temperatures [36,38,39,40,41]. Long storage time duration of deli meats at retail or at home under improper temperature control may significantly increase the risk. In addition, cross-contamination from the retail environment may increase the risk in the unopened products [36]. The results of a survey showed that the most listeriosis cases of RTE cooked meat sliced at retail stores related to a long storage of the products and high temperatures of domestic refrigerators [40]. The temperature distributions recorded in this study appear to have high variability in most of the checked refrigerators. In many cases, the periodic increase in temperatures is probably due to the defrosting system of the refrigerators [34,42]. Other factors that cause high variability in product temperatures are the influence of lighting and nearby standing customers, as well as the interruptions of the cold chain by resupplying the refrigerators [42].

The logistics of the supply chain of RTE products includes storage at the production point or distribution centers, transportation, and retail and domestic storage. The temperatures during the first stages of the chain in most cases are properly controlled [36]. On the other hand, the products in retail stores are out of the manufacturer’s control and often deviate from the regulated temperature limits. The storage temperatures in retail stores vary depending on factors such as the type of refrigerator and the product’s position inside the refrigerators [36]. In the present study, we found a high percentage of stores that kept up-to-date with the records of the storage temperatures. The stores also had good cleanliness conditions on the refrigerator shelves, grocery shelves, and storage room. Moreover, in the storage room, we noticed improvement after the A′ audit. Lundén, Vanhanen, Kotilainen, et al. reported that in food retail stores, the lowest conformity of own-check documentation occurred in the monitoring of refrigerator temperatures; furthermore, in most of the stores, the documentation of the refrigerator temperature was not sufficient [22]. In the same study, non-conformities in cleanliness occurred in facilities out of the sight of the consumers, such as the storage rooms, the facilities for the handling of slicing meat, and the cleaning equipment rooms [22]. Another study indicated that the retailers were unaware of the importance of maintaining the cold chain and, combined, with the lack of a control system during storage in stores, temperatures were not recorded [42]. In the present study, the frequent phenomenon was that the stores stored “Spoiled food products/products for return” beneath the fresh ones in the same refrigerator (Figure 3B). In addition, it was observed that spoiled products or products for return were placed in bags or upside down at the bottom of the selling refrigerator of fresh products. The most common justification made by the employees was the lack of space or that there is not a non-compliance since the products are separated. We also noticed a low conformity in checking the temperatures of receiving products. The most common cause was the lack of information or the lack of time of the store’s employees, but in some cases, it was also the non-cooperation of the raw materials’ distributor to carry out inspections in the vehicle. Similar results were observed in another study [19], in which approximately 25% of food service and retail stores of leafy greens stored raw material beneath cooked food in the same refrigerators, increasing the risk of cross-contamination. Moreover, researchers have noticed many cases of non-compliance with implications for food safety, such as the improper use of thermometers, lack of documentation processes, and poor employee hygiene. In the same study and in accordance with our observations, it was also noticed that no temperatures of the receiving products were recorded [19]. Implementing prerequisite programs (PRPs) and a HACCP system is often challenging for businesses, especially SMEs. Despite the fact that the principles of PRPs and HACCP have been in place for many years, implementing an effective FSMS is difficult for some FBs. The effective implementation of an FSMS depends on overcoming a combination of managerial, organizational, and technical obstacles. Large FBs with big financial resources are more likely to invest in food safety rather than smaller FBs [28].

Another important factor that affects the implementation of effective FSMSs is the personnel experience. Any lack of knowledge of food safety principles inevitably leads to weakness in the developed system, so the proper implementation of PRPs and HACCP requires training [28]. The food handlers may not recognize themselves as one of the most significant links in food safety, and they are convinced that the other handlers were worse [43]. All the non-compliances reported previously were correlated with a lack of awareness of food safety risks and could potentially be attributed to the lack of knowledge and training in food safety [44]. Several reports in the literature indicate that training programs had a positive impact on knowledge and contributed to significant improvement in food safety [43,45,46,47]. There is a need to change wrong behaviors related to hygiene that can be achieved through a systematic approach, the regular training of employees, and continuous active surveillance of the personal hygiene and handling techniques of employees [24]. Wu, Burnett, et al. [25] suggest that management should create an environment in which employees are motivated to identify themselves as important members of the food safety program.

## 5. Conclusions

The purpose of this study was to investigate whether there would be any substantial improvements between two consecutive audits in a single chain of stores, so the audit results were limited in this specific food retail chain of stores. The decrease in the number of clusters for any given parameter between audits A′ and B′ suggests that an effect took place regarding implementation of the corrective actions. However, many of the food retail stores did not fully comply with all food safety requirements. One of the most interesting findings is that the employees of the stores did not check and record the temperature of receiving products from suppliers. A poor hygiene practice was also the storage of spoiled products beneath fresh products for selling in the same refrigerator. Most of the time when this happened, it was accompanied by another incorrect practice, i.e., not monitoring and recording the temperatures of the products for selling. An important factor to comply with the food hygiene principles is correlated to the will of personnel to implement them despite the difficulties, as well as the creation of a food safety culture. This highlights the importance of continuous monitoring and improvement in food safety and management systems to maintain high standards in food retail stores. Future research could build upon the findings of this study by exploring the factors that contribute to the implementation and effectiveness of food safety and management systems in food retail stores. A better understanding of these factors may lead to the development of more effective strategies for improving food safety and management in food retail chain stores in Greece and beyond.

## Figures and Tables

**Table 1:** Statistical analysis of the percentage of refrigerators with temperatures above the critical ones between audits A′ and B′.

Refrigerator Shelf	Critical Temperature (°C)	Percentages of Stores at A′ Audit	Percentages of Stores at B′ Audit	*p*-Value
Fruit refrigerator	8 °C	15%	8%	>0.05
Salad refrigerator	5 °C	51%	39%	<0.05
Yogurts and desserts refrigerator	3 °C	44%	38%	>0.05
Milk refrigerator	6 °C	16%	17%	>0.05
Cheese refrigerator	5 °C	16%	19%	>0.05
Deli meat refrigerator	3 °C	48%	44%	>0.05
Fish/Smoked fish refrigerator	4 °C	18%	13%	>0.05
Meat refrigerator	4 °C	18%	13%	>0.05
Poultry refrigerator	4 °C	20%	13%	>0.05

## Data Availability

Data available on request.

## Supplementary Materials

The following supporting information can be downloaded at: https://www.mdpi.com/article/10.3390/foods13030457/s1, Table S1: Temperatures occurring in the two cycles of audits.
