# Supplementary material for: Food Safety and Management System Audits in Food Retail Chain Stores in Greece
Source: Foods. 2024 Feb 1;13(3):457. doi: 10.3390/foods13030457 (PMC10855910; doi:10.3390/foods13030457)
Supplement: Supplementary file 1 [file foods-13-00457-s001.zip › foods-2784314-supplementary.pdf]

Table S1. Temperatures occurred in the two cycles of audits

Temperature (^oC)	A′ audit				B′ audit			
	Median	IQR	Min	Max	Median	IQR	Min	Max
Fruit store refrigerator	5.1	3	-2	13	5	2.5	0	13
Salads refrigerator	5.25	3	-2	13	5	3	0	13
Yogurts & desserts refrigerator	3	3	-4	15	2.8	3.25	0	13
Milk refrigerator	4	4	-2	17	3	4	-2	15
Cheese refrigerator	3	3.1	-1.7	15	3	3	0	13
Deli meat refrigerator	3	3.2	-1	13	3	3	0	13
Fish/Smoked Fish refrigerator	2	3.25	-7	16	1	2.7	-0.3	11
Meat refrigerator	2	3.5	-7	19	1	2.7	-0.3	11
Poultry refrigerator	2	3.5	-7	19	1	2.7	-0.3	11
